# The Calcilytic Agent NPS 2143 Rectifies Hypocalcemia in a Mouse Model With an Activating Calcium-Sensing Receptor (CaSR) Mutation: Relevance to Autosomal Dominant Hypocalcemia Type 1 (ADH1)

**DOI:** 10.1210/en.2015-1269

**Published:** 2015-06-08

**Authors:** Fadil M. Hannan, Gerard V. Walls, Valerie N. Babinsky, M. Andrew Nesbit, Enikö Kallay, Tertius A. Hough, William D. Fraser, Roger D. Cox, Jianxin Hu, Allen M. Spiegel, Rajesh V. Thakker

**Affiliations:** Academic Endocrine Unit (F.M.H., G.V.W., V.N.B., M.A.N., E.K., R.V.T.), Radcliffe Department of Medicine, Oxford Centre for Diabetes, Endocrinology and Metabolism, University of Oxford, Oxford, OX3 7LJ, United Kingdom; Medical Research Council (MRC) Mammalian Genetics Unit and Mary Lyon Centre (T.A.H., R.D.C.), MRC Harwell, Harwell Science and Innovation Campus, Oxfordshire, OX11 0RD, United Kingdom; Department of Medicine (W.D.F.), Norwich Medical School, University of East Anglia, Norwich, NR4 7TJ, United Kingdom; Laboratory of Bioorganic Chemistry (J.H.), National Institute of Diabetes and Digestive and Kidney Diseases, Bethesda, Maryland 20892; and Albert Einstein College of Medicine (A.M.S.), Bronx, New York 10461

## Abstract

Autosomal dominant hypocalcemia type 1 (ADH1) is caused by germline gain-of-function mutations of the calcium-sensing receptor (CaSR) and may lead to symptomatic hypocalcemia, inappropriately low serum PTH concentrations and hypercalciuria. Negative allosteric CaSR modulators, known as calcilytics, have been shown to normalize the gain-of-function associated with ADH-causing CaSR mutations in vitro and represent a potential targeted therapy for ADH1. However, the effectiveness of calcilytic drugs for the treatment of ADH1-associated hypocalcemia remains to be established. We have investigated NPS 2143, a calcilytic compound, for the treatment of ADH1 by in vitro and in vivo studies involving a mouse model, known as *Nuf*, which harbors a gain-of-function CaSR mutation, Leu723Gln. Wild-type (Leu723) and *Nuf* mutant (Gln723) CaSRs were expressed in HEK293 cells, and the effect of NPS 2143 on their intracellular calcium responses was determined by flow cytometry. NPS 2143 was also administered as a single ip bolus to wild-type and *Nuf* mice and plasma concentrations of calcium and PTH, and urinary calcium excretion measured. In vitro administration of NPS 2143 decreased the intracellular calcium responses of HEK293 cells expressing the mutant Gln723 CaSR in a dose-dependent manner, thereby rectifying the gain-of-function associated with the *Nuf* mouse CaSR mutation. Intraperitoneal injection of NPS 2143 in *Nuf* mice led to significant increases in plasma calcium and PTH without elevating urinary calcium excretion. These studies of a mouse model with an activating CaSR mutation demonstrate NPS 2143 to normalize the gain-of-function causing ADH1 and improve the hypocalcemia associated with this disorder.

Autosomal dominant hypocalcemia type 1 (ADH1) and ADH2 (Online Mendelian Inheritance in Man [OMIM] numbers 601198 and 615361) are caused by germline gain-of-function mutations of the calcium-sensing receptor (CaSR) and G protein subunit α-11 (1–4), respectively, which play a pivotal role in the parathyroid and renal regulation of extracellular calcium (Ca^2+^_o_) concentrations. Gain-of-function CaSR mutations have been demonstrated to induce biased signaling responses that involve the preferential activation of phospholipase C-mediated intracellular calcium (Ca^2+^_i_) mobilizations (Figure 1) (5), which lead to decreased PTH secretion and increased urinary calcium excretion (2, 4, 6). ADH1-associated mutations may also enhance CaSR biosynthesis by stabilizing newly formed CaSRs in an active conformation that protects against proteasomal degradation (7, 8).

**Figure 1.**
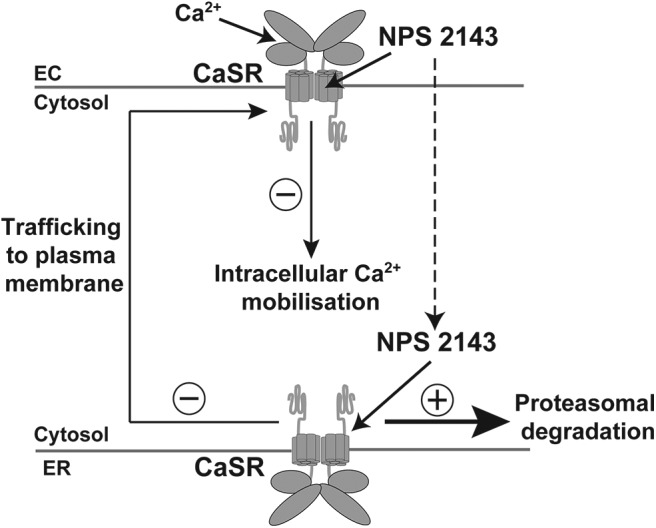
Schematic representation of the mechanism of action of NPS 2143. NPS 2143 binds to the TMD of plasma membrane-expressed CaSRs and decreases Ca^2+^_o_-mediated signaling responses such as Ca^2+^_i_ mobilization. Prolonged exposure of CaSR-expressing cells to NPS 2143 may lead to the internalization of this calcilytic compound, which is considered to bind to newly formed CaSRs within the endoplasmic reticulum (ER), and destabilize their active conformation, thus leading to protein misfolding and proteasomal degradation (8), which would in turn diminish the pool of receptors available for trafficking to the plasma membrane. EC, extracellular.

Approximately 50% of patients with ADH1 develop hypocalcemic symptoms such as paraesthesia, carpo-pedal spasms, and seizures (1, 3, 9–12). Although ADH1 is associated with increased circulating phosphate concentrations and inappropriately low or normal PTH concentrations, this is considered to represent a distinct disease entity from hypoparathyroidism, because affected individuals generally have PTH concentrations that are detectable and may either be below or within the reference range (1, 9, 10), and also a relative hypercalciuria that is characterized by urinary calcium to creatinine ratios that are within or above the reference range (1, 9). Ectopic calcification of the kidneys and basal ganglia is a common feature of ADH1 and affects more than 35% of patients (1, 10, 12). Patients with CaSR mutations that lead to a severe gain-of-function may also develop a Bartter-like syndrome characterized by hypokalemic alkalosis, renal salt wasting, and hyperreninemic hyperaldosteronism (11, 13, 14). Active vitamin D metabolites, combined with adequate dietary calcium intake and/or use of calcium supplements, are currently the mainstay of treatment for symptomatic ADH1 patients. However, their use predisposes affected individuals to the development of marked hypercalciuria, nephrocalcinosis, nephrolithiasis, and renal impairment (1, 9).

Compounds that selectively bind to the CaSR and allosterically inhibit the function of this G protein-coupled receptor represent a potential targeted therapy for ADH (15–18). Indeed, these negative allosteric CaSR modulators, which are known as calcilytics, have been demonstrated, in vitro, to improve the gain-of-function associated with ADH-causing CaSR mutations (19–22). The mechanism of action of calcilytic drugs involves binding to plasma membrane CaSRs and diminishing receptor signaling responses in the presence of orthosteric agonist (20). Moreover, prolonged exposure of cells expressing gain-of-function mutant CaSRs to calcilytic drugs may facilitate internalization of these allosteric modulators, which then bind and destabilize the conformation of nascent CaSRs, thus leading to enhanced proteasomal degradation of mutant receptors (Figure 1) (7, 8). However, some gain-of-function mutations located within the CaSR transmembrane domain (TMD), which is predicted to be the binding site for allosteric CaSR modulators (23, 24), have been shown to impair the effectiveness of calcilytic drugs (Supplemental Figure 1) (19–22, 25). Moreover, it is unclear whether calcilytic drugs may improve the hypocalcemia of ADH1 while minimizing the risk of hypercalciuric renal disease. We have assessed the effectiveness of a phenylalkylamine calcilytic compound, known as NPS 2143, for the treatment of ADH1 using a mouse model, known as *Nuf*, which harbors a germline nonconstitutively activating mutation, Leu723Gln, located within the second intracellular loop of the CaSR TMD (Supplemental Figure 1) (26). *Nuf* mice were originally identified for having opaque flecks in the nucleus of the lens and have an ADH phenotype characterized by hypocalcemia, hyperphosphatemia, inappropriately reduced plasma PTH concentrations, and ectopic calcification (26). Before embarking on an in vivo assessment of the efficacy of the calcilytic NPS 2143 in rectifying the *Nuf* mouse hypocalcemia, we used first an in vitro functional assay to determine whether NPS 2143 may normalize the gain-of-function associated with the *Nuf* Leu723Gln mutation, because some TMD-located ADH-associated CaSR mutations have been reported to be unresponsive or partially responsive to calcilytic drugs (19–22, 25).

## Materials and Methods

### In vitro assessment of NPS 2143 on the functional responses of the Leu723Gln mutant CaSR

Wild-type and mutant CaSR-pEGFP-N1 constructs were generated as described previously (26) and transiently transfected into HEK293 cells using Lipofectamine Plus (Invitrogen), as described (2, 26). The wild-type and mutant CaSRs were functionally assessed by measuring alterations in Ca^2+^_i_ concentrations in response to changes in Ca^2+^_o_ concentrations, as described previously (2, 26). Forty-eight hours after transfection, the cells were harvested, washed in calcium- and magnesium-free Hanks' balanced salt solution (Invitrogen), and loaded with 1-μg/mL indo-1-acetoxymethylester (Molecular Probes) for 1 hour at 37°C. After removal of free dye, the cells were resuspended in 1 mL of calcium- and magnesium-free Hanks' balanced salt solution and maintained at 37°C. Transfected HEK293 cells were incubated with either a 15% aqueous solution of 2-hydroxypropyl-β-cyclodextrin (Sigma) (vehicle) or NPS 2143 for one hour at the following concentrations: 0nM, 20nM, 40nM, and 80nM. Flow-assisted cell sorting was performed with a Cytomation MoFlo flow cytometer (Dako-Cytomation) equipped with an argon laser (Coherent Radiation), as described (26). Baseline fluorescence ratio was measured for 2 minutes, the fluorescence ratio vs time was recorded, and data were collected for 2 minutes at each Ca^2+^_o_ concentration. Cytomation summit software was used to determine the peak mean fluorescence ratio of the transient response after each individual stimulus expressed as a normalized response, as previously reported (26, 27). The EC_50_ (ie, Ca^2+^_o_ concentration required for 50% of the maximal response) for each normalized concentration-response curve was determined.

### In vivo administration of NPS 2143 to Nuf mice

*Nuf* mice were maintained on the inbred 102/H background, which is a substrain bred at the Mary Lyon Centre. Mice were kept in accordance with United Kingdom Home Office welfare guidance and project license restrictions. Mice were fed ad libitum on a commercial diet (which contained 1.2% calcium, 0.8% phosphate, and 3000-IU/kg vitamin D; Rat and Mouse Diet No. 3; Special Diet Services). A single bolus of NPS 2143 or vehicle (15% aqueous solution of 2-hydroxypropyl-β-cyclodextrin) was administered by ip injection to wild-type, *Nuf*/+, and *Nuf/Nuf* mice aged between 16 and 20 weeks (n = 8–14 for all groups). An aqueous solution of 2-hydroxypropyl-β-cyclodextrin was used as the drug vehicle for NPS 2143, because this has previously been demonstrated to be effective at dissolving this calcilytic compound and to be safe and well tolerated in rodent studies (15, 16, 28). Based on the findings of previous rodent studies involving NPS 2143 (16, 28), a dose of 30 mg/kg was administered to wild-type and *Nuf* mice. Plasma samples were obtained at either 0, 1, 4, or 24 hours by tail vein or terminal bleed. Urine samples were obtained from untreated mice or mice that had been bled at 1 hour after administration of NPS 2143 or drug vehicle alone and then immediately housed in metabolic cages (Tecniplast) for a 24-hour period and fed ad libitum on water and powdered chow (29). Urine samples were analyzed for calcium and creatinine and plasma samples analyzed for total calcium, albumin, sodium, potassium, phosphate, urea, creatinine, and alkaline phosphatase activities using a Beckman Coulter AU680 analyzer, as previously described (29). Plasma calcium was adjusted for albumin using the formula: ((albumin-mean albumin) × 0.02) + calcium, as reported (29). The calcium to creatinine clearance ratio (CCCR) was calculated using the formula U_Ca_/P_Ca_ × P_Cr_/U_Cr_, where U_Ca_ is the urinary concentration of calcium in mmol/L, P_Ca_ is the plasma concentration of adjusted calcium in mmol/L, U_Cr_ is the urinary concentration of creatinine in mmol/L, and P_Cr_ is the plasma concentration of creatinine in mmol/L (29). Plasma PTH concentrations were determined using an ELISA for mouse intact PTH (Immutopics), as described (30).

### Statistical analyses

For the in vitro functional expression studies, the mean EC_50_ from 4 separate transfection experiments was used for statistical comparison by using the Mann-Whitney *U* test. For the in vivo studies, the data from male and female mice were pooled for analysis, because no statistically significant differences were observed between these groups. A Mann-Whitney *U* test was used to compare biochemical and metabolic variables between wild-type and affected *Nuf* mice, in which the Bonferroni correction for multiple testing was applied. *P* < .05 was considered significant for all analyses.

## Results

### Effect of the calcilytic NPS 2143 on the function of the mutant Leu723Gln CaSR

**Figure 2.**
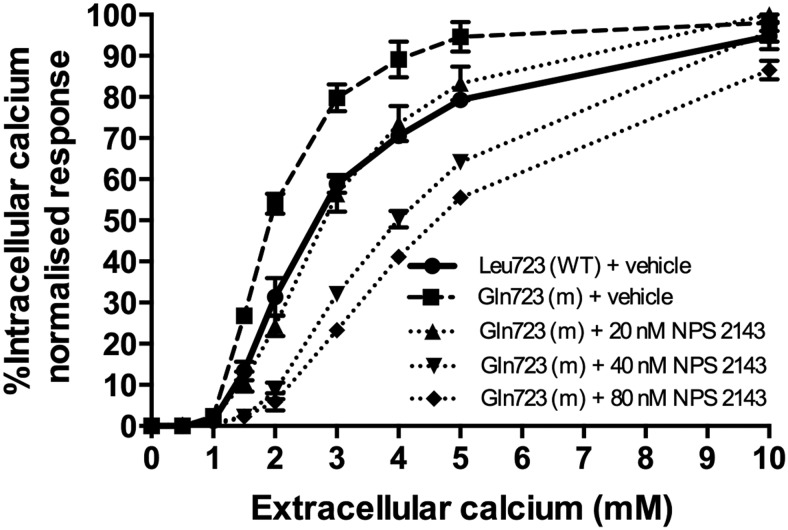
Effect of NPS 2143 on the concentration-response curve of the mutant Gln723 CaSR in transfected HEK293 cells. HEK293 cells were transiently transfected with wild-type or the mutant CaSR-EGFP construct. Single, live cells were loaded with indo-1-acetoxymethylester, which emits fluorescence at 525 nm. NPS 2143 was added at 0nM, 20nM, 40nM, and 80nM concentrations to HEK293 cells transfected with the mutant Gln723 CaSR-EGFP. Cells transfected with the CaSR were selected by fluorescence-activated cell sorting, and the Ca^2+^_o_-evoked increases in Ca^2+^_i_ concentrations were measured. The concentration-response curves of the untreated (dashed line) and NPS 2143-treated (dotted lines) mutant Gln723 receptor were compared with the untreated wild-type (WT) Leu723 CaSR-EGFP (solid line). The increments in Ca^2+^_o_ concentrations from 0mM to 10mM are shown on the x-axis, and the Ca^2+^_i_ response, which was measured as a percentage of the maximum normalized response, is shown on the y axis (mean ± SEM of 4 estimations).

The responses of wild-type and mutant CaSRs to alterations in Ca^2+^_o_ concentrations were assessed, after transient transfection of HEK293 cells, by measurements of Ca^2+^_i_ concentrations (Figure 2). In agreement with previous findings (26), the mutant Gln723 CaSR showed a significant leftward shift in its concentration-response curve when compared with the wild-type Leu723 CaSR, thereby demonstrating that the mutant CaSR is activated by a lower [Ca^2+^]_o_ than the wild type, consistent with this leading to a gain of CaSR function. Indeed, the mutant Gln723 CaSR had a significantly (*P* < .01) reduced EC_50_ (1.94 ± 0.07mM) when compared with the EC_50_ of the wild type (2.53 ± 0.14mM) (Figure 2 and Table 1). A dose titration of the calcilytic agent, NPS 2143, in HEK293 cells expressing the mutant Gln723 CaSR revealed that NPS 2143 at a concentration of 20nM led to a rightward shift of the mutant receptor concentration-response curve (EC_50_ of 2.79 ± 0.19, *P* = .33 compared with wild type) (Table 1), so that this was indistinguishable to that of the wild-type Leu723 CaSR (Figure 2), and thus the function of the mutant receptor was normalized. The addition of higher doses of NPS 2143 (40nM and 80nM) led to a marked rightward shift of the concentration-response curve, so that the mutant Gln723 CaSR displayed a loss-of-function with significantly raised EC_50_ values of more than or equal to 4.0mM (*P* < .01) (Figure 2 and Table 1).

**Table 1.** EC_50_ Values for Wild-Type Leu723 and Mutant Gln723 CaSRs in the Presence of Different NPS 2143 Concentrations

CaSR Construct	EC_50_ (mM)			
	Mean Value	SEM	n	*P* Value (vs WT)
Leu723 (WT) + vehicle	2.53	±0.14	4	—
Gln723 (m) + vehicle	1.94	±0.07	4	<.01
Gln723 (m) + 20nM NPS 2143	2.79	±0.19	4	.33
Gln723 (m) + 40nM NPS 2143	3.97	±0.10	4	<.01
Gln723 (m) + 80nM NPS 2143	4.56	±0.09	4	<.01

The number (n) of separate transfection experiments is indicated. WT, wild type; m, mutant.

### Effect of the calcilytic NPS 2143 on the hypocalcemia of *Nuf* mice

**Figure 3.**
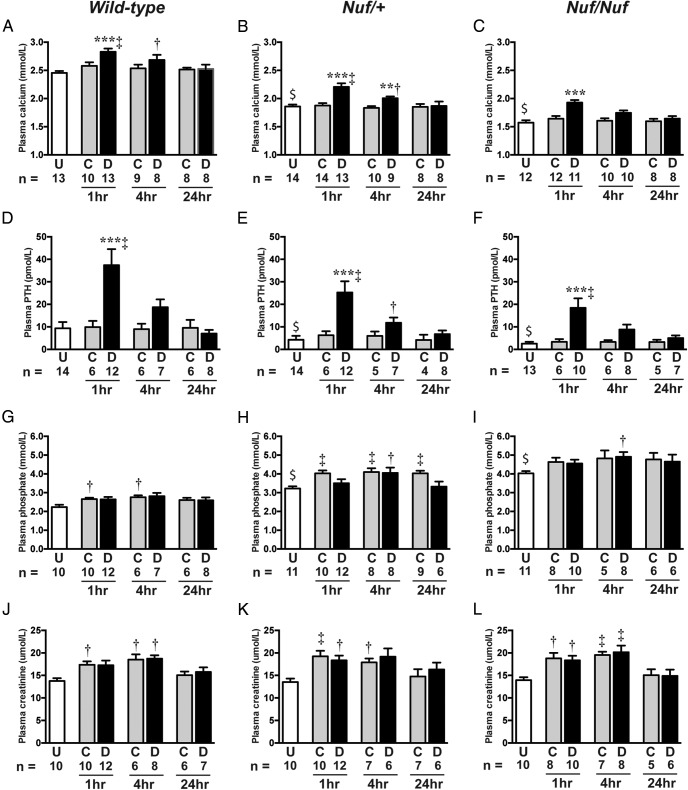
Plasma adjusted calcium, PTH, phosphate, and creatinine concentrations after ip injection of either NPS 2143 or drug vehicle. Plasma concentrations of (A–C) adjusted calcium, (D–F) PTH, (G–I) phosphate, and (J–L) creatinine were measured in untreated (U) mice, or 1, 4, or 24 hours after mice were given an ip bolus injection of control (C) or drug (D) solutions (n = 4–14 for all groups). $, *P* < .001 for a comparison between untreated (U) *Nuf*/+ or *Nuf/Nuf* mice and respective untreated wild-type mice. **, *P* < .01 and ***, *P* < .001 for a comparison between respective wild-type, *Nuf*/+, or *Nuf/Nuf* mice given control (C) or drug (D) solutions. †, *P* < .05 and ‡, *P* < .01 for a comparison between wild-type, *Nuf*/+, or *Nuf/Nuf* mice given control (C) or drug (D) solutions and respective untreated (U) mice.

The in vitro studies revealed that NPS 2143 was effective in rectifying the gain-of-function of the mutant Gln723 CaSR, and we therefore pursued studies to determine whether ip injection of the calcilytic compound NPS 2143 could improve the hypocalcemia associated with *Nuf* mice. Untreated *Nuf*/+ and *Nuf/Nuf* mice were significantly hypocalcemic and hyperphosphatemic (*P* < .001), and had significantly reduced plasma PTH concentrations when compared with age-matched wild-type mice (Figure 3). No significant alterations were noted in plasma concentrations of sodium, potassium, creatinine, and alkaline phosphatase activity between wild-type and affected *Nuf* mice (Table 2 and Supplemental Tables 1 and 2). Intraperitoneal bolus administration of NPS 2143 resulted in a significant (*P* < .001) rise in plasma calcium concentrations at 1 hour after injection in wild-type, *Nuf*/+, and *Nuf/Nuf* mice (Table 2, Supplemental Tables 1 and 2, and Figure 3, A–C). Thus, NPS 2143 successfully improved the hypocalcemia associated with *Nuf*/+ and *Nuf/Nuf* mice compared with mice given the drug vehicle alone or untreated mice. At 4 hours after NPS 2143 administration, plasma calcium values remained significantly elevated in wild-type and affected *Nuf*/+ mice compared with respective untreated mice (Figure 3, A and B), and at 24 hours, plasma calcium concentrations decreased to levels that were not significantly different from mice given the drug vehicle alone or untreated mice (Figure 3, A–C). Furthermore, NPS 2143 treatment resulted in a marked rise in plasma PTH concentrations in wild-type and affected *Nuf* mice at 1 hour (Figure 3, D–F). PTH concentrations decreased to baseline values at 24 hours after NPS 2143 administration (Figure 3, D–F). Treatment with NPS 2143 did not significantly alter plasma phosphate concentrations in wild-type or affected *Nuf* mice compared with control group mice (Table 2, Supplemental Tables 1 and 2, and Figure 3, G–I). Indeed, administration of NPS 2143 or the drug vehicle alone was associated with significant increases in plasma phosphate in wild-type and affected *Nuf* mice at 1 and 4 hours compared with respective untreated mice (Figure 3, G–I), and these increases in plasma phosphate were accompanied by elevations in plasma urea and creatinine concentrations (Table 2 and Figure 3, J–L), which may indicate dehydration leading to renal impairment. NPS 2143 administration was otherwise well tolerated and not associated with adverse effects. A single ip dose of NPS 2143 had no significant effect on urinary calcium parameters such as 24-hour urinary calcium excretion, calcium to creatinine ratio, or CCCR (Table 3 and Supplemental Tables 3 and 4).

**Table 2.** Effect of NPS 2143 Treatment on Plasma Biochemical Parameters

Parameters	Genotypes								
	Wild Type			*Nuf*/+			*Nuf/Nuf*		
	Untreated	Vehicle Only	Drug	Untreated	Vehicle Only	Drug	Untreated	Vehicle Only	Drug
Sodium (mmol/L)	146 ± 0.7	147 ± 0.7	146 ± 0.6	145 ± 0.6	146 ± 0.7	148 ± 0.6	146 ± 0.7	146 ± 0.8	146 ± 0.7
	(n = 12)	(n = 7)	(n = 8)	(n = 12)	(n = 8)	(n = 7)	(n = 11)	(n = 8)	(n = 8)
Potassium (mmol/L)	4.9 ± 0.1	4.8 ± 0.2	4.9 ± 0.1	4.7 ± 0.2	4.8 ± 0.1	4.7 ± 0.2	4.5 ± 0.2	4.9 ± 0.1	4.8 ± 0.2
	(n = 10)	(n = 8)	(n = 8)	(n = 12)	(n = 7)	(n = 7)	(n = 11)	(n = 7)	(n = 8)
Urea (mmol/L)	11.3 ± 0.7	12.4 ± 0.6^e^	12.9 ± 0.6^e^	11.6 ± 0.6	13.7 ± 0.6^e^	13.5 ± 0.5^e^	11.9 ± 0.6	14.3 ± 0.7^e^	14.3 ± 0.7^e^
	(n = 10)	(n = 10)	(n = 12)	(n = 11)	(n = 9)	(n = 12)	(n = 12)	(n = 8)	(n = 10)
Creatinine (μmol/L)	14.6 ± 0.9	17.4 ± 0.7^c^	17.3 ± 1.0	13.6 ± 0.7	19.2 ± 1.2^d^	18.3 ± 1.0^c^	13.9 ± 0.5	18.8 ± 1.2^c^	18.3 ± 1.0^c^
	(n = 10)	(n = 10)	(n = 12)	(n = 10)	(n = 10)	(n = 12)	(n = 10)	(n = 8)	(n = 10)
Albumin (g/L)	31.1 ± 0.5	29.7 ± 0.5	29.7 ± 0.4	29.8 ± 0.7	28.9 ± 0.5	28.3 ± 0.5	29.8 ± 0.4	28.4 ± 0.6	28.5 ± 0.5
	(n = 13)	(n = 10)	(n = 13)	(n = 14)	(n = 14)	(n = 14)	(n = 13)	(n = 12)	(n = 11)
Calcium (mmol/L)	2.50 ± 0.06	2.58 ± 0.06	2.83 ± 0.06^b^	1.87 ± 0.04^a^	1.88 ± 0.04	2.21 ± 0.06^b^	1.62 ± 0.05^a^	1.64 ± 0.05	1.93 ± 0.04^b^
	(n = 13)	(n = 10)	(n = 13)	(n = 14)	(n = 14)	(n = 13)	(n = 12)	(n = 12)	(n = 11)
Phosphate (mmol/L)	2.21 ± 0.13	2.66 ± 0.07^c^	2.64 ± 0.13	3.22 ± 0.11^a^	4.03 ± 0.15^d^	3.50 ± 0.2	4.03 ± 0.12^a^	4.63 ± 0.22	4.55 ± 0.2
	(n = 10)	(n = 10)	(n = 12)	(n = 11)	(n = 10)	(n = 12)	(n = 11)	(n = 8)	(n = 10)
ALP (U/L)	131 ± 7.1	128 ± 7.4	128 ± 8.3	121 ± 7.3	122 ± 8.1	142 ± 8.8	123 ± 7.2	118 ± 5.0	147 ± 15
	(n = 10)	(n = 10)	(n = 12)	(n = 11)	(n = 11)	(n = 12)	(n = 10)	(n = 10)	(n = 12)
PTH (pmol/L)	9.4 ± 2.8	9.9 ± 2.8	37.4 ± 7.1^b^	4.3 ± 1.8^a^	6.3 ± 1.8	25.3 ± 5.0^b^	2.6 ± 0.8^a^	3.4 ± 1.2	18.4 ± 4.2^b^
	(n = 14)	(n = 6)	(n = 12)	(n = 14)	(n = 6)	(n = 12)	(n = 13)	(n = 6)	(n = 10)

Plasma biochemical values were measured in untreated mice and at 1 hour after administration of NPS 2143 or drug vehicle only. All values are expressed as mean ± SEM. Plasma calcium concentrations were normalized to the mean plasma albumin concentration. ALP, alkaline phosphatase activity.

a *P* < .001 for a comparison between untreated *Nuf*/+ or *Nuf/Nuf* mice and respective untreated wild-type mice.

b *P* < .001 for a comparison of mice given NPS 2143 vs respective mice given drug vehicle only.

c *P* < .05 for a comparison between wild-type, *Nuf*/+, or *Nuf/Nuf* mice given vehicle only or NPS 2143 and respective untreated mice.

d *P* < .01 for a comparison between wild-type, *Nuf*/+, or *Nuf/Nuf* mice given vehicle only or NPS 2143 and respective untreated mice.

e *P* < .05 before Bonferroni correction for a comparison between wild-type, *Nuf*/+, or *Nuf/Nuf* mice given vehicle only or NPS 2143 and respective untreated mice.

**Table 3.** Effect of NPS 2143 Treatment on Urinary Calcium Parameters

Parameters	Genotypes								
	Wild Type			*Nuf*/+			*Nuf/Nuf*		
	Untreated	Vehicle Only	Drug	Untreated	Vehicle Only	Drug	Untreated	Vehicle Only	Drug
24-h Ca	3.4 ± 0.8	3.6 ± 1.0	3.8 ± 0.9	2.6 ± 0.3	2.0 ± 0.2	2.7 ± 0.6	2.4 ± 0.5	2.4 ± 0.2	1.9 ± 0.4
	(n = 8)	(n = 7)	(n = 7)	(n = 7)	(n = 6)	(n = 7)	(n = 7)	(n = 6)	(n = 6)
Ca/Cr	0.31 ± 0.05	0.28 ± 0.04	0.23 ± 0.02	0.29 ± 0.05	0.24 ± 0.05	0.20 ± 0.02	0.24 ± 0.03	0.18 ± 0.02	0.15 ± 0.03
	(n = 8)	(n = 7)	(n = 7)	(n = 7)	(n = 6)	(n = 7)	(n = 7)	(n = 6)	(n = 6)
CCCR	0.0017 ± 0.0002	0.0019 ± 0.0002	0.0014 ± 0.0002	0.0019 ± 0.0005	0.0024 ± 0.0005	0.0018 ± 0.0002	0.0020 ± 0.0002	0.0022 ± 0.0004	0.0013 ± 0.0002
	(n = 8)	(n = 7)	(n = 7)	(n = 7)	(n = 6)	(n = 7)	(n = 7)	(n = 6)	(n = 6)

Urinary calcium excretion (24-h Ca) and urinary calcium to creatinine ratio (Ca/Cr) were measured using urine samples obtained over a 24-hour period from untreated mice or from mice administered NPS 2143 or drug vehicle alone. The CCCR was measured using plasma samples obtained at 1 hour, and urine samples were obtained over a 24-hour period from untreated mice or from mice administered NPS 2143 or drug vehicle alone. Urinary calcium excretion values are shown as μmol/24 hours. No significant differences in urinary calcium parameters were observed between untreated mice and mice given the drug vehicle or between NPS 2143-treated mice and mice given the drug vehicle alone. All values are expressed as mean ± SEM.

## Discussion

The calcilytic compound, NPS 2143, was assessed as a potential targeted therapy for ADH1 by studies involving *Nuf* mice, which harbor a gain-of-function Leu723Gln CaSR mutation (26). NPS 2143 was used as it induces more prolonged elevations in circulating PTH concentrations than other calcilytic drugs (17, 31) and has been shown to be safe and well tolerated in rodent studies (15). However, previous in vitro studies have indicated that NPS 2143 may not be a suitable therapy for all gain-of-function CaSR mutations (19–22, 25). Indeed, ADH-associated CaSR mutations, such as Ala835Asp and Ala843Glu, which are located close to the Glu837 TMD residue that is critical for NPS 2143 binding (Supplemental Figure 1) (19, 21, 32), have been demonstrated to completely abrogate the action of NPS 2143, whereas ADH-associated TMD CaSR mutations located away from known NPS 2143 binding residues, such as Glu767Gln, Leu773Arg and Asn802Ile, may partially diminish the efficacy of this calcilytic compound (Supplemental Figure 1) (20, 22, 25). We therefore evaluated the effects of NPS 2143, in vitro, on the *Nuf* Leu723Gln mutation, which is also located in the TMD (Supplemental Figure 1), before assessing its efficacy in vivo. Our use of a cell-based assay that functionally expressed the Leu723Gln CaSR mutant, revealed the gain-of-function associated with the Leu723Gln mutation to be wholly responsive to NPS 2143. This calcilytic acted in a dose-dependent manner and restored the Leu723Gln mutant EC_50_ to a value that was indistinguishable to the wild-type receptor, thereby normalizing the sensitivity of the CaSR to Ca^2+^_o_. Indeed, the *Nuf* Leu723Gln mutation responded to nanomolar concentrations of NPS 2143, whereas previous in vitro studies of CaSR mutations leading to ADH, indicate that micromolar concentrations of this calcilytic drug may be required to rectify associated signal transduction abnormalities (19, 20, 22). The responsiveness of the Leu723Gln mutation to low doses of NPS 2143 may be explained by the finding that this mutation is not constitutively activating and induced less than 25% reduction in the EC_50_ value compared with the wild-type CaSR, whereas CaSR mutations leading to ADH1 generally cause a more than 30% reduction in the EC_50_ value (1, 19, 21, 22, 25). Thus, such in vitro assays that characterize the function of mutant CaSRs may provide a personalized medicine approach to the selection of appropriate targeted therapies for specific ADH1-causing CaSR mutations. Such targeted therapies may include phenylalkylamines or the quinazolinone class of calcilytic compounds that have also recently been shown to normalize the gain-of-function associated with NPS 2143-resistant ADH1 mutations in vitro (21) and have potential for the management of patients harboring such mutations.

In support of these findings demonstrating the in vitro efficacy of NPS 2143 for the *Nuf* mouse Leu723Gln CaSR mutation, in vivo administration of this calcilytic led to 4- to 5-fold increases in plasma PTH concentrations in both wild-type and affected *Nuf* mice, which is in keeping with a reported study involving wild-type rats (16). These elevations in plasma PTH were accompanied by a 0.25–0.30 mmol/L increase in plasma calcium values in wild-type, *Nuf*/+, and *Nuf/Nuf* mice and indicate that gene dosage did not impact on drug efficacy. Moreover, the amelioration of hypocalcemia in *Nuf* mice was not associated with any increase in urinary calcium excretion, which typically occurs when ADH1 patients are treated with active vitamin D metabolites (1, 9). Indeed, NPS 2143 may be expected to lower urinary calcium excretion, which has been reported in wild-type rat studies (28). The *Nuf* mice urine biochemical parameters were assessed using 24-hour urine samples, and any reduction in urinary calcium excretion may not have been apparent if the renal effects of a single dose of NPS 2143 lasted less than 24 hours. A longer-term study that involves repetitive dosing and a period of acclimatization to the metabolic cage environment may be required to more accurately determine the effects of NPS 2143 on urinary calcium excretion (29). The duration of action of ip injected NPS 2143 on plasma calcium and PTH concentrations was short-lived compared with oral administration of this drug. Indeed, delivery of NPS 2143 to wild-type rats by oral gavage has been previously shown to result in prolonged elevations of circulating PTH concentrations that lasted more than 4 hours (15, 28), whereas plasma PTH had decreased by 4 hours after ip injection in the present study. Of note, NPS 2143 administration did not lead to a lowering of plasma phosphate concentrations, despite inducing a marked rise in plasma PTH. Although an observed lack of a phosphate-lowering effect of NPS 2143 may be consequence of the blood sampling time points, plasma phosphate concentrations were actually shown to increase after administration of NPS 2143 or the drug vehicle alone. The most likely cause of the hyperphosphatemia was a decrease in renal function that occurred in mice receiving the drug or control solutions. Although ip injection represents a widely used method of drug delivery in rodents, this drug administration route may be associated with increased physiological stress (33), thereby leading to reduced fluid intake and dehydration. In keeping with studies involving wild-type rats (28), NPS 2143 had a selective action on plasma calcium and PTH but did not influence other biochemical parameters such as plasma sodium, potassium or creatinine. However, the present study involved single dose administration of NPS 2143, and repetitive dosing studies are required to confirm the efficacy of calcilytic compounds in inducing sustained increases in circulating calcium and PTH concentrations in the setting of ADH1 and to determine whether these CaSR-targeted drugs may also ameliorate the hyperphosphatemia and hypercalciuria associated with this disorder.

In summary, these studies involving *Nuf* mice with an activating CaSR mutation demonstrate that NPS 2143-mediated allosteric inhibition of the CaSR is able to rectify the molecular defect underlying ADH1 and also improve the reduced plasma calcium and PTH levels that are associated with this disorder. These findings indicate that the calcilytic NPS 2143, or related compounds, have potential as targeted therapies for the hypocalcemia associated with ADH1.
